# Essential hypertension: A filtered serum based metabolomics study

**DOI:** 10.1038/s41598-017-02289-9

**Published:** 2017-05-19

**Authors:** Keerti Ameta, Ashish Gupta, Sudeep Kumar, Rishi Sethi, Deepak Kumar, Abbas Ali Mahdi

**Affiliations:** 1 Department of Biochemistry, King George’s Medical University, Lucknow, India; 2 Centre of Biomedical Research, SGPGIMS Campus, Lucknow, India; 3 Department of Cardiology, SGPGIMS, Lucknow, India; 4 Department of Cardiology, King George’s Medical University, Lucknow, India

## Abstract

Despite the easy and reliable methods of blood pressure measurement, the screening of essential hypertension (EH) is usually ignored due to delayed onset of symptoms. A probe into the biochemical changes in hypertension would serve as a welcome asset to provide insight into the mechanistic aspects of EH. Filtered serum samples from 64 EH patients and 59 healthy controls (HC) were analysed using 800 MHz nuclear magnetic resonance (NMR) spectroscopy. Application of principal component analysis (PCA) and orthogonal partial least-squares discriminant analysis (OPLS-DA) following receiver operating characteristic (ROC) curve of NMR data reveals significantly perturbed metabolites: alanine, arginine, methionine, pyruvate, adenine, and uracil. This set of metabolites correctly classified 99% of cases from HC and also showed excellent correlation in both isolated elevated diastolic blood pressure (DBP) cases and combined elevated systolic-diastolic blood pressure cases. Proton NMR metabolomics of EH may prove helpful in defining associated biomarkers and serve as an alternate diagnostic tool with judicious clinical assessment.

## Introduction

Hypertension had a global burden on 26.4% of the adult population in 2000, and projections reveal a rise to 29.2 per cent by 2025^1^. In India, the rising burden of hypertension is evident from an increase in prevalence from 5% to 20–40% in the past three decades, which is indeed alarming^2, 3^. The etiology of 90% of the cases of hypertension remains unclear and is termed as essential hypertension (EH)^4^. Given its high prevalence and associated risks of progression to cardio-vascular disease (CVD) and stroke, early diagnosis becomes crucial.

Despite the presence of easy and reliable blood pressure evaluation methods (using manual or automated sphygmomanometer), screening of hypertension is usually ignored because of the late appearance of symptoms. Moreover, EH pathophysiology is not merely restricted to elevated blood pressure. Factors such as lifestyle, environmental influences, and disturbances in vascular structure play an important role in EH. The genetic relationship has been largely explored^5, 6^; however, the perturbations in metabolic and biochemical pathways in EH remain less explored. The use of metabolomic strategies for probing the metabolic aspects of EH may improve our understanding of altered biochemical pathways.

Experimental studies using urine samples of genetically hypertensive rats suggest a close association between NMR profiles and perturbed metabolism^7, 8^. Studies have addressed the issue of searching and probing into essential hypertension using proton NMR spectroscopy, but they either represented preliminary data^9^ or represented sub-types of hypertension^10^ using either intact serum or plasma samples obtained from human subjects. The use of intact serum and plasma samples might have resulted in masking of valuable information. So we evolved a novel filtered serum (lacking lipoproteins and proteins) based metabolomic approach with three fold aims: (1) to obtain qualitative information from proton NMR data in filtered serum samples of EH and age comparable healthy controls (HC); (2) to quantify those metabolites that caused differences between the two study groups, (3) to create a prediction model to confirm our results using multivariate statistical analysis.

## Results

**Figure 1:**
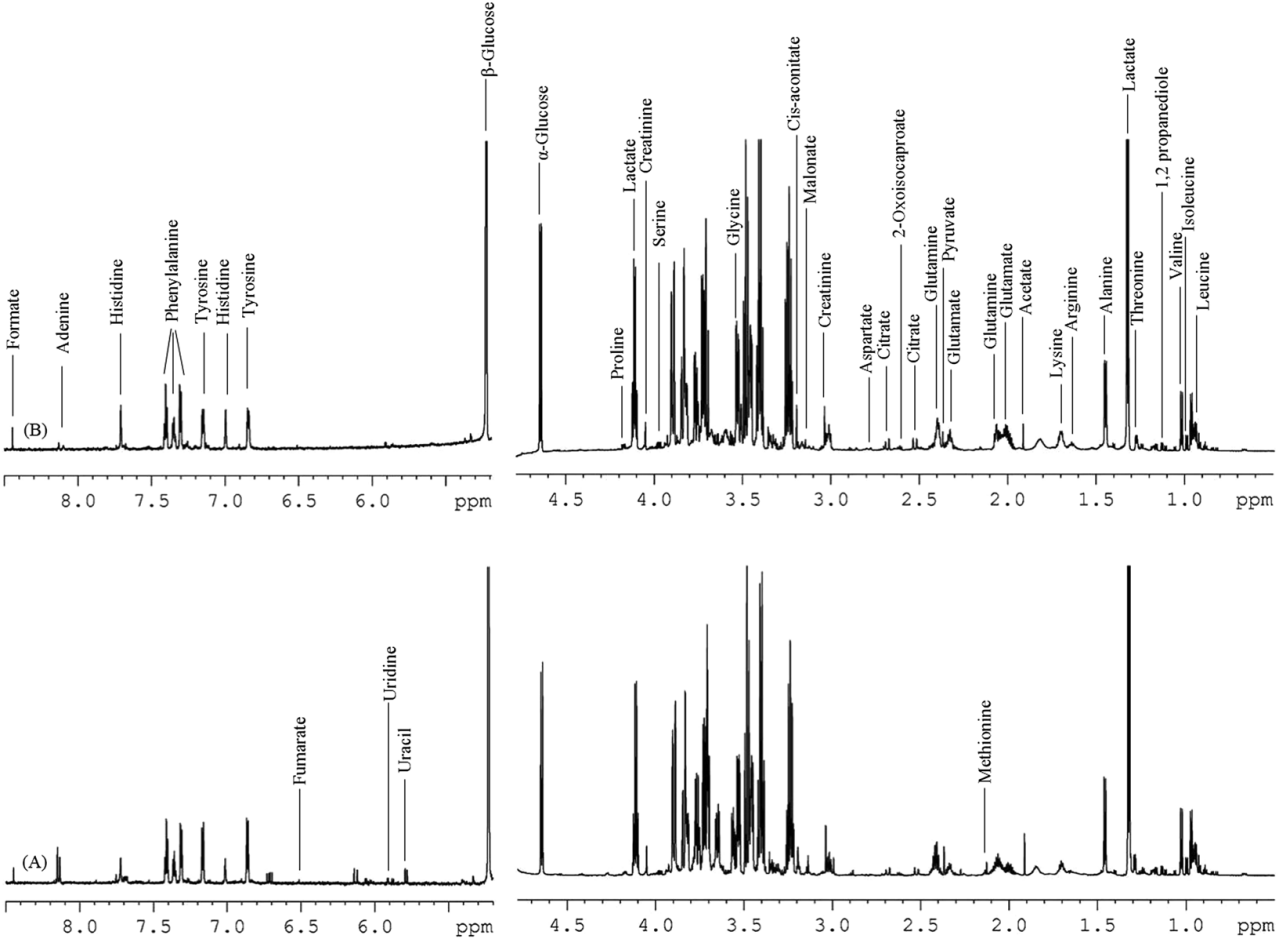
A typical ^1^H NMR spectrum of serum samples after removal of proteins and lipoproteins (**A**) healthy control (**B**) essential hypertension, showing assignment of various metabolites.

A detailed one dimensional ^1^H NMR spectrum of EH and HC is shown in Fig. 1. The demographic and clinical data of all study subjects are summarised in Table 1.

**Table 1:** Study subjects characteristics. Unpaired t test was applied between EH and HC subjects.

S. No.		EH Cases (n = 64)	HC Subjects (n = 59)	
1.	Age (years)	43 ± 6	42 ± 5	*p* = 0.305
2.	Sex	Male	Male	
3.	Stage I Hypertension	45 (70.31%)	0%	
	(a) Isolated elevated DBP	31 (68.8%)	0%	
	(1) SBP (mm Hg)	132.43 ± 9.56	120.67 ± 2.44	*p* = 0.0001
	(2) DBP (mm Hg)	95.45 ± 2.51	79 ± 2.09	*p* = 0.0001
	(b) Combined elevated SBP-DBP	14 (31.1%)	0%	
	(1) SBP (mm Hg)	151.57 ± 3.89	120.67 ± 2.44	*p* = 0.0001
	(2) DBP (mm Hg)	95.07 ± 2.92	79 ± 2.09	*p* = 0.0001
4.	Stage II Hypertension	19 (29.6%)	0%	
	(a) Isolated elevated DBP	0%	0%	
	(1) SBP (mm Hg)	NA	NA	
	(2) DBP (mm Hg)	NA	NA	
	(b) Combined elevated SBP-DBP	19 (100%)	0%	
	(1) SBP (mm Hg)	164.68 ± 3.36	120.67 ± 2.44	*p* = 0.0001
	(2) DBP (mm Hg)	105.42 ± 3.37	79 ± 2.09	*p* = 0.0001
5.	Total isolated elevated DBP cases	31 (48.4%)	0%	
6.	Total combined elevated SBP-DBP cases	33 (51.5%)	0%	
7.	Serum creatinine (mg/dl)	0.91 ± 0.17	0.85 ± 0.21	*p* = 0.078
8.	Blood urea nitrogen (mg/dl)	14.6 ± 3.1	13.6 ± 2.1	*p* = 0.064
9.	Glycosylated Hb (%)	5.6 ± 0.3	5.6 ± 0.2	*p* = 0.052
10.	Na^+^ (mEq/L)	141 ± 4	139 ± 5	*p* = 0.060
11.	K^+^ (mEq/L)	3.9 ± 0.5	4.1.0 ± 0.4	*p* = 0.062
12.	Haematocrit (%)	45.8 ± 2.7	47.0 ± 3.2	*p* = 0.105
13.	Homocyst(e)ine	11.4 ± 4.8	7.2 ± 1.3	*p* = 0.0001
14.	Lipid profile			
	(a) Total Cholesterol	200.72 ± 33.8	188.3 ± 41.1	*p* = 0.080
	(b) Triglyceride	137.06 ± 21.2	123.5 ± 19.1	*p* = 0.0002
	(c) High density lipoprotein (HDL)	37.7 ± 3.2	41.4 ± 4.7	*p* = 0.0001
	(d) Low density lipoprotein (LDL)	135.5 ± 35.9	123 ± 42	*p* = 0.068
	(e) Very low density lipoprotein (VLDL)	27.4 ± 4.2	24.7 ± 3.8	*p* = 0.0002
15.	Family history of EH	43 (67.1%)	21 (35.5%)	*p* = 0.0003
16.	Smoking (before enrolment)	38 (59.3%)	31 (52.5%)	*p* = 0.450

### Multivariate statistical analysis

**Figure 2:**
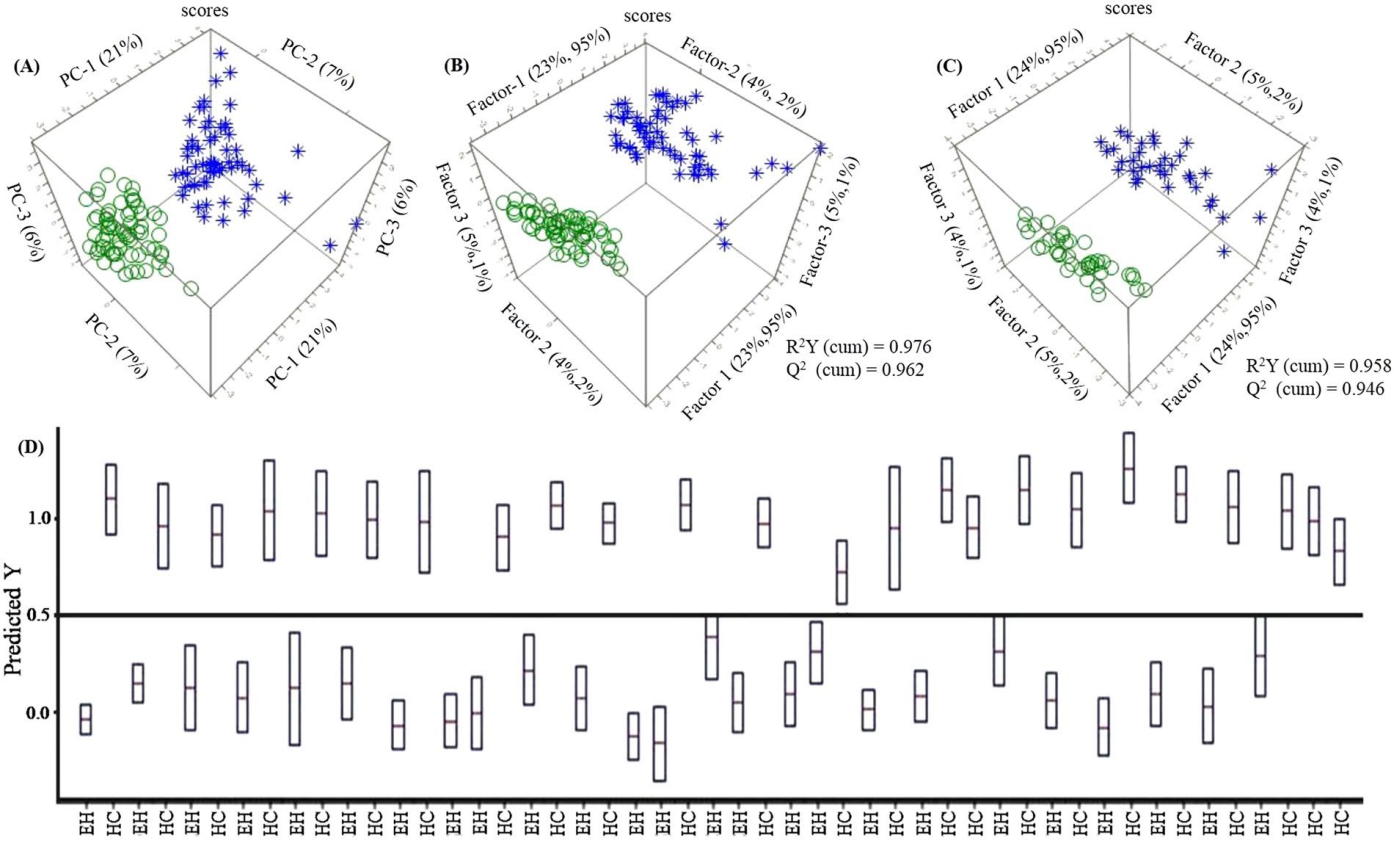
Three-dimensional score plots of (**A**) unsupervised PCA (**B**) supervised OPLS-DA (**C**) OPLS-DA of training data and (**D**) Y-prediction of test data. Here; green color circles denote EH; blue color stars denote HC.

First of all, AMIX-generated binned data were used for PCA analysis and to construct the PCA score plot between EH and HC cohorts (Fig. 2). The scatter plot of PCA exhibited that EH cohorts were well clustered and separated from HC cohorts (Fig. 2A), based on the milieu of ailment.

An OPLS-DA approach was executed to achieve a next level objective statistical analysis. The outcomes revealed a well-separated, narrowly clustered pattern between EH and HC score plot (Fig. 2B). Since the OPLS-DA approach applied cohort information to generate utmost segregation between different cohorts, this advanced analysis determines profound and putative biomarkers relevant to particular cohorts. The robustness of the OPLS-DA model is further explained by excellent values of presentation statistics, R^2^Y and Q^2^, exhibited in Fig. 2B.

To avoid any errors of the mathematical model, the OPLS-DA was again executed on the training and test data set with a leave-one-out approach. This ICV process exhibits the determination of the model in the form of excellent R^2^ and Q^2^ values a corresponding OPLS-DA score plot of training data (Fig. 2C) and Y-predicted test data (Fig. 2D). The outcomes validate the OPLS-DA assessment for a possible novel method to probe EH and segregate from HC with minimal invasiveness. The comparable precision of the training and test data of the OPLS-DA suggest that the use of minimally invasive filtered serum metabolomics exerting NMR spectroscopy is very promising to determine EH.

### Selection of Biomarkers

Assessment of ^1^H NMR spectra revealed the plethora of metabolites. The human metabolome database (http://www.hmdb.ca) and various recently published studies^11–13^ were used to assign the metabolites such as different types of amino acids, nucleic acids, monosaccharides, energetic related molecules (pyruvate, lactate, citrate, creatinine) as well as amines and choline’s present in EH and HC filtered serum samples.

**Figure 3:**
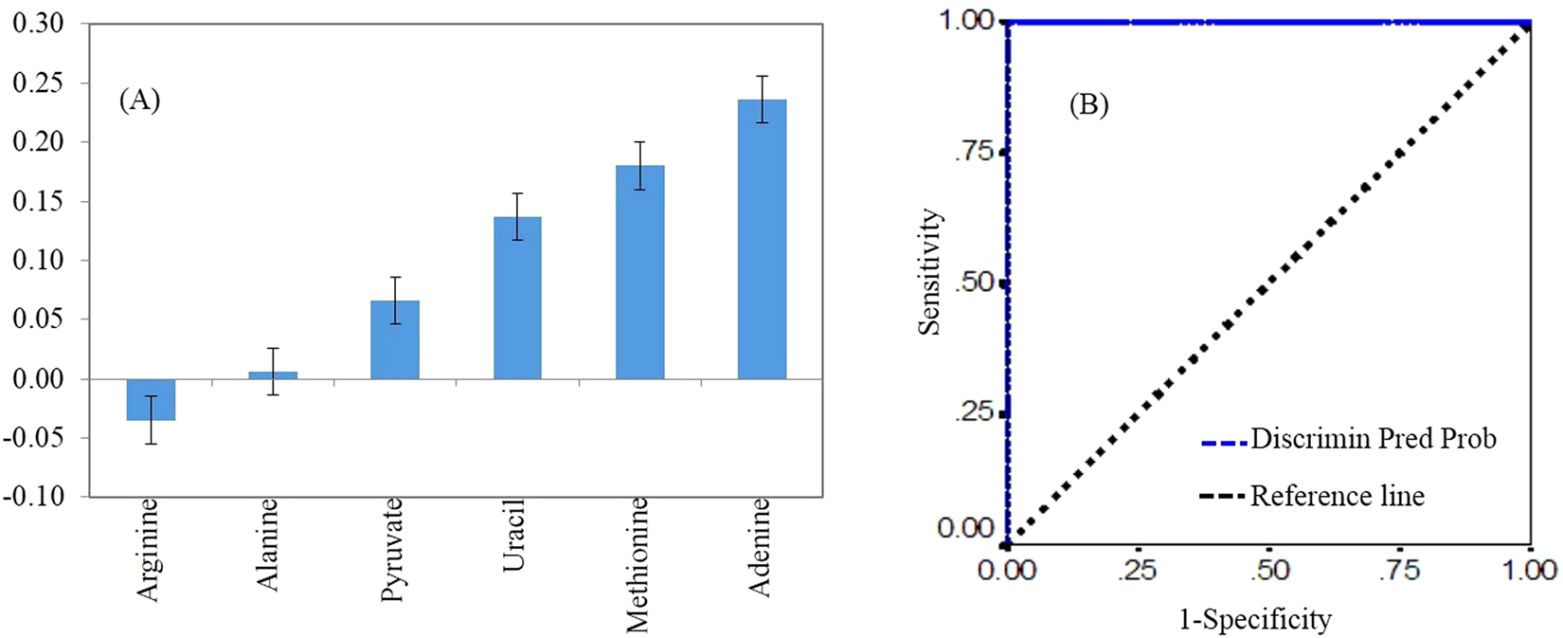
(**A**) Alterations in OPLS-DA coefficients (±SE) of filtered serum metabolites from EH patients as compared to HC. (**B**) Defines receiver operating characteristic (ROC) curve obtained from a combination of the six metabolites. Discrimin Pred Prob; Discriminant predicted probability.

The following several steps screening approach were cautiously carried out to determine potential biomarkers: (a) OPLS-DA loading plot reveals the profound altered bins from the metabolic profile of HC and EH following retrospective assignment of metabolites using ^1^H NMR based metabolic content. (b) The signature metabolites for the differentiation of EH from HC cohorts are personified by the OPLS-DA derived coefficients. (c) Spectral analysis of filtered serum reveals that among the plethora of metabolites, only six metabolites (alanine, pyruvate, methionine, arginine, adenine, and uracil) were playing a significant role to segregate and classify EH from HC cohorts. (d) The subsequent analysis demonstrated up-regulation of arginine and down-regulation of alanine, pyruvate, methionine, adenine, and uracil in EH compared to HC. The coefficient values with standard error of these variables are shown in Fig. 3A.

The next level evaluation is the endorsement of appropriately reaped latent biomarkers for their clinical significance. We achieved this evaluation with the help of ROC analysis.The ROC curve of a combination of these six variables depicts the classification of EH from HC subjects with a sensitivity of 99%, specificity of 99%, and an area under the curve (AUC) of 99% (Fig. 3B). On the basis of thoughtfully opted these latent biomarkers, and ROC evaluation suggested that EH equated to HC not only generate signature metabolites, but also that these metabolites may be applied to discriminate them. Hence, these metabolites were considered as the most decisive metabolites for distinguishing between EH and HC.

**Figure 4:**
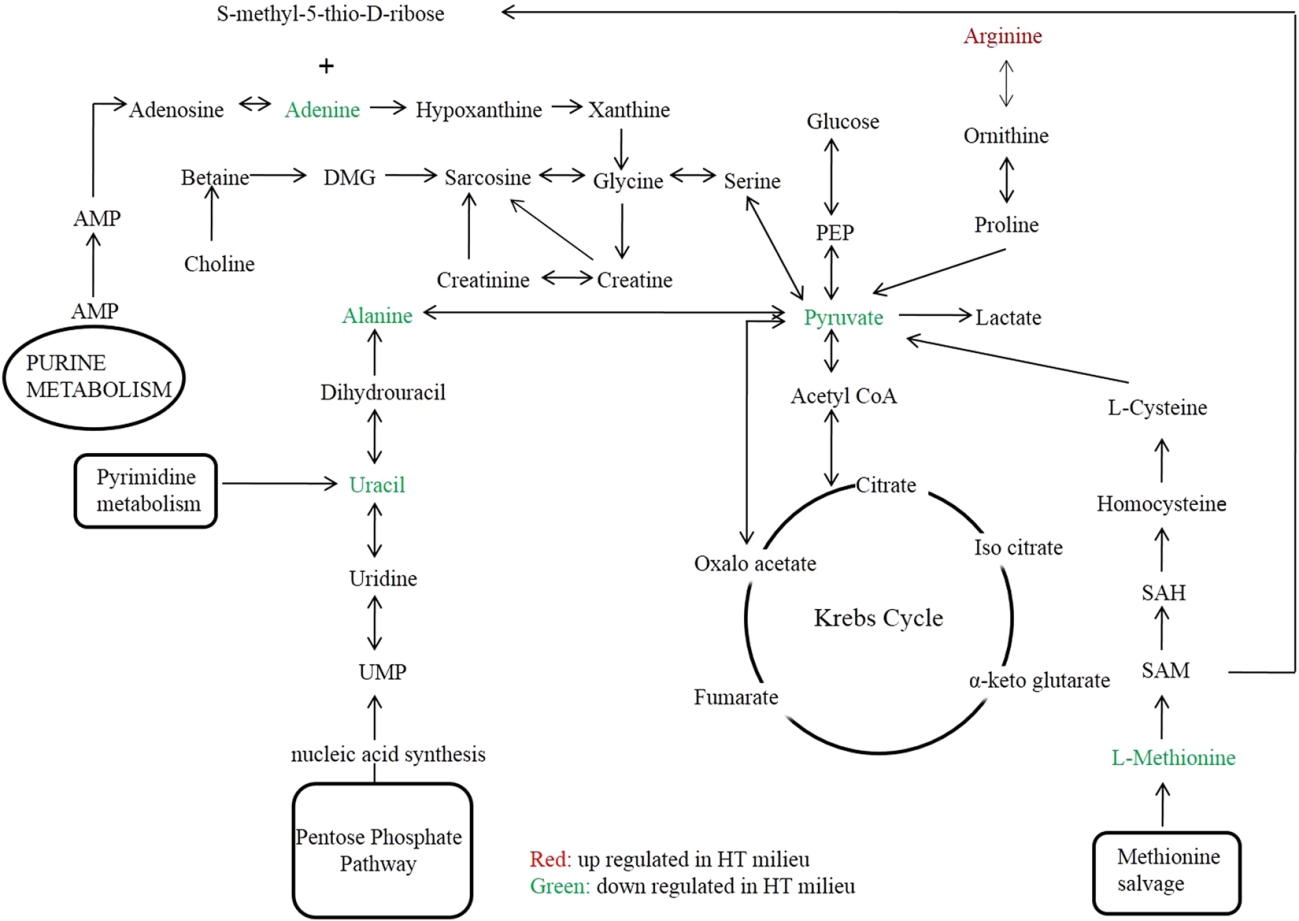
A simplified metabolic pathway showing up-regulated and down-regulated metabolites in EH patients as compared to HC.

The perturbed metabolism has been shown in a simple metabolic pathway (Fig. 4).

Regression analysis of serum metabolomics derived six biomarkers with diastolic blood pressure revealed excellent correlation in the group of isolated DBP: DBP vs. alanine (*R* ^*2*^ = 0.35), DBP vs. arginine (*R* ^*2*^ = 0.56), DBP vs. methionine (*R* ^*2*^ = 0.64), DBP vs. pyruvate (*R* ^*2*^ = 0.78), DBP vs. adenine (*R* ^*2*^ = 0.77), and DBP vs. uracil (*R* ^*2*^ = 0.73) (Figure S1).

A separate linear regression analysis was performed in patients with combined elevated SBP-DBP (14 stage I and 19 stage II hypertensive patients). The correlation values were: SBP vs. alanine (*R* ^*2*^ = 0.39), SBP vs. arginine (*R* ^*2*^ = 0.50), SBP vs. methionine (*R* ^*2*^ = 0.68), SBP vs. pyruvate (*R* ^*2*^ = 0.77), SBP vs. adenine (*R* ^*2*^ = 0.78), and SBP vs. uracil (*R* ^*2*^ = 0.76) (Figure S2) while DBP vs. alanine (*R* ^*2*^ = 0.38), DBP vs. arginine (*R* ^*2*^ = 0.50), DBP vs. methionine (*R* ^*2*^ = 0.59), DBP vs. pyruvate (*R* ^*2*^ = 0.69), DBP vs. adenine (*R* ^*2*^ = 0.73), and DBP vs. uracil (*R* ^*2*^ = 0.69) (Figure S3).

## Discussion

The principal finding of our study is that the ^1^H NMR metabolomics technique presented distinct qualitative features in circulating metabolites of filtered serum samples of EH and HC. Secondly, the relative amount of perturbed metabolites significantly differ in EH and HC cohorts. Last, the set of six metabolites created marked separation between study subjects and correctly classified 99% of patients with high sensitivity and specificity. Moreover, these variables demonstrated excellent linear correlations with SBP and DBP measures. Despite relevant evidence from previous studies about serum metabolic biomarkers of hypertension, to the best of our knowledge, ours is the first report that has highlighted metabolic perturbations in filtered serum samples from EH and HC and that resulted in elaboration of less abundant metabolites.

The etiology of essential hypertension is affected by several factors. The manifestation of elevated blood pressure levels comes from complex interactions among genetic, environmental, and lifestyle factors. A variety of factors including oxidative stress, endothelial dysfunction, vascular tone, salt sensitivity, the sympathetic nervous system (SNS) involvement, and the renin angiotensin aldosterone system (RAAS)^14–19^ influence systemic arterial blood pressure.

We observed an increase in serum arginine levels of EH patients compared to HC. This may primarily be attributable to the dynamic flux of arginine and the retarded transport of L-arginine via y+ transporters^20^. Also, the hemodynamics of L-arginine in serum is a representative of the complex interplay/crosstalk between its synthesis via arginosuccinate and lyase and the degradation via arginase, that is, the arginase activity being altered in humans and experimental models of hypertension^21–23^. In contrast to the previous studies where they measures targeted metabolites, this study has witnessed an actual translation of arginine-NO mechanism and their effect on other important metabolites as well using a single snap-shot metabolic profile of EH individuals.

A change in L-arginine/NO pathway has been proposed as a causative factor of hypertension^24^. However, we have recognized practical implication of the same. L-arginine is well known for its vasodilating affect mediated by endothelial derived nitric oxide synthase in mammals^25^. However, this tells only a part of the story. An important factor that needs consideration is arginine paradox^26^. Arginine paradox is a phenomenon where on one hand; arginine supplementation improves NO mediated vasodilation^27^; while the arginine reserves remain above the Michelis-Menten constant, k_m_ for nitric oxide synthase^28, 29^. An important limitation of earlier studies is either they have been carried in animal models or healthy individuals and this study explore the actual mechanism relating to EH pathology in humans. A number of explanations were later on sought to answer the arginine paradox including altered arginase^30^, LDL mediated impaired L-arginine transport to endothelial cells^31^, compartmentalization of L-arginine^32^ and involvement of NOS inhibitors like asymmetric dimethyl arginine (ADMA)^33^ and monomethyl arginine (L-NMMA). Although all these studies supported the idea of involvement of perturbed arginine levels in hypertension, the findings came either from animal model^30^, cell-lines^31, 32^ or a limited number of patients with certain co-morbid condition^33^. As far as our results are concerned, the most relevant explanation seems to be the involvement of ADMA^23, 34^. ADMA operates by inhibiting competitively for NO synthase, thereby decreasing NO bioavailability, despite high L-arginine concentrations^23^. ADMA is released from hydrolysis of post-translationally methylated proteins^35^. It is assumed that the reduced activity of the enzyme dimethylarginine dimethylaminohydrolase (DDAH) results in elevated ADMA concentration^35^. Attenuation of DDAH activity is influenced by factors like oxidized low-density lipoprotein cholesterol^36^, inflammatory cytokines, hyperhomocysteinemia^37^ and hyperglycemia^38^, thereby causing accumulation of ADMA and subsequent reduced NO synthesis. Moreover, plasma ADMA levels have been reported to be increase in hypertensive individuals^39, 40^. ADMA has been shown in experimental and clinical studies to play an important role in perturbing arterial pressure^41, 42^. Also, determining L-arginine to ADMA ratio has been advocated^35^. These previous findings originated from either experimental model of hypertension^41^, EH patients with positive exercise testing (an indication of underlying CAD)^39^ or limited number of EH patients^40^. In contrast, the outcome of this study is actually transcending these phenomena in considerable number of EH patients against HC using judiciously high level of statistical appraisal.

In addition, nitric oxide itself works in many dimensions. Apart from the conventional vasodilation property, the reaction of NO with reactive oxygen species leads to formation of peroxynitrite formation^43, 44^. This complies from the fact that essential hypertension is also affected by impaired oxygen^14^ and nitrogen species^45^. These diverse involvements may further diminish NO bioavailability. Moreover, the combined action of NO and peroxynitrite radicals have been reported for diverse actions on lipids, DNA and held responsible for a variety of pathogenic mechanisms^46, 47^. Even though these studies indicate the role of NO in various disease, the findings of the present study are actual depictors of what might be happening in EH cases.

Increased insulin levels as a result of insulin resistance also play a dynamic role in L-arginine and NO pathway. Insulin signalling causes release of NO^48^ and aids in vasodilation to decrease blood pressure. Still, the bioavailability of NO is determined by mutual contrasting factors whereby arginine promotes insulin release^49^ from beta cells of pancreas and insulin stimulates arginine cellular uptake resulting in NO mediated vasodilation^50^.

Another fact that requires a special mention in context of the results of this study is the NO mediated inhibition of methionine synthase. Both *in vitro* ^51^ and *in vivo* ^52^, methionine synthase has been shown to be inhibited, thus restricting methionine biosynthesis. This is yet another evidence of importance of methionine as a key descriptor in EH pathology worked out from human samples. Methionine is sulphur containing essential amino acid. A decreased level of methionine with a corresponding increase in homocysteine levels in EH patients as compared to HC may reflect a trend towards increased conversion of methionine to homocysteine which agrees with earlier findings establishing the role of homocysteine in aggravating hypertension mediated by diminished NO bioavailability^53, 54^. Also, methionine supplementation has been proved to exhibit vasoconstriction^55^, thereby providing an indirect proof that methionine near absence levels in EH patients’ NMR profiles is a reflection of manifested high blood pressure. While these studies offer relevant insights into undergoing mechanism with their inherent limitations. Either they involve cultured cells^53^ or involve experimental models of hypertension^54^ or are results from pilot study^55^. Here, the present study offer a clear picture of elevated levels of homocysteine and consequent diminished levels of methionine in EH patients, thus justifying their role as bio-signatures. Moreover, evidence of increased S-adenosyl-homocysteine (SAH) and decreased S-adenosyl methionine (SAM) levels in hypertensive patients’ support to the outcome of NMR derived metabolomics^56^. These perturbed levels of SAM and SAH indicate switching off methionine salvage pathways to adenine metabolism, which is indicated in the NMR spectra of EH subjects in accordance with KEGG pathways (M00034; map 00270). A perturbation in adenine levels has also been supported by its role in precipitating essential hypertension and aortic stiffness^57^. The previous observations narrated using the experimental model of hypertension^57^; however, in contrast, the comparable observations reveals using NMR derived translational nature of metabolomics in identifying metabolites peculiar of EH individuals.

L-alanine shows a diminishing effect on release of nor-epinephrine from cardiac sympathetic nerves in animal models, resulting in blood pressure elevation^58^. This effect is mediated by alpha receptors of norepinephrine. Thus, modulation of cardiovascular responses and circulating catecholamines seems responsible for alanine’s role in blood pressure elevation^59^. Apart from this, L-alanine’s interaction with insulin also influences arterial blood pressure^60^. The insulinotropic effect of L-alanine is reported to operate either via co-transport of Na^+^ ions and increased ATP production^61^ or Ca^2+^ion-uptake^62^. Thus, L-alanine consumption leads to insulin release, thereby affecting blood pressure.

Moreover, urinary alanine levels have been linked with hypertension in INTERMAP epidemiological study^63^. This indeed is a reflection that alanine biochemistry is an integral part of human physiology and may be controlling the hypertension through aspects like impaired insulin resistance^64^ or imbalance between renal transport and transport along the muscle-liver axis^65^. Although conflicting evidence exists relating the levels of alanine aminotransferase in hypertensive patients^66, 67^, the decreased level of alanine along with a parallel decrease in pyruvate levels in EH compared to HC may be a manifestation of a dynamic balance between aminotransferase reaction in muscle and liver. Moreover, the decline in pyruvate levels has been supported in previous study^68^ conducted on a limited number of hypertensive individuals. This further explains role of insulin resistance at the site of muscles in manifestation of EH. Apart from this, the lactate-to-pyruvate ratio is increasing in EH patients compared to HC, which again agrees with earlier findings in cases of moderate intra-abdominal hypertension and benign hypertension^69, 70^. Our observations reveals an inherent uniqueness since alanine and pyruvate levels have been described as playing important role in manifestation of EH, unlike its sub types or variants.

Uracil has been shown to possess an antihypertensive property, and the near absence of this in EH patients may follow from this property^71^. This again is an observation from cultured cells and supports our plausible mechanism. However, the actual translation from perspective of cultured cells to human system is the distinctive feature of our results. Urapidil is a derivative of uracil that has also been used as an antihypertensive drug^72^, exhibits the connection of uracil in perturbed metabolism during essential hypertension.

**Figure 5:**
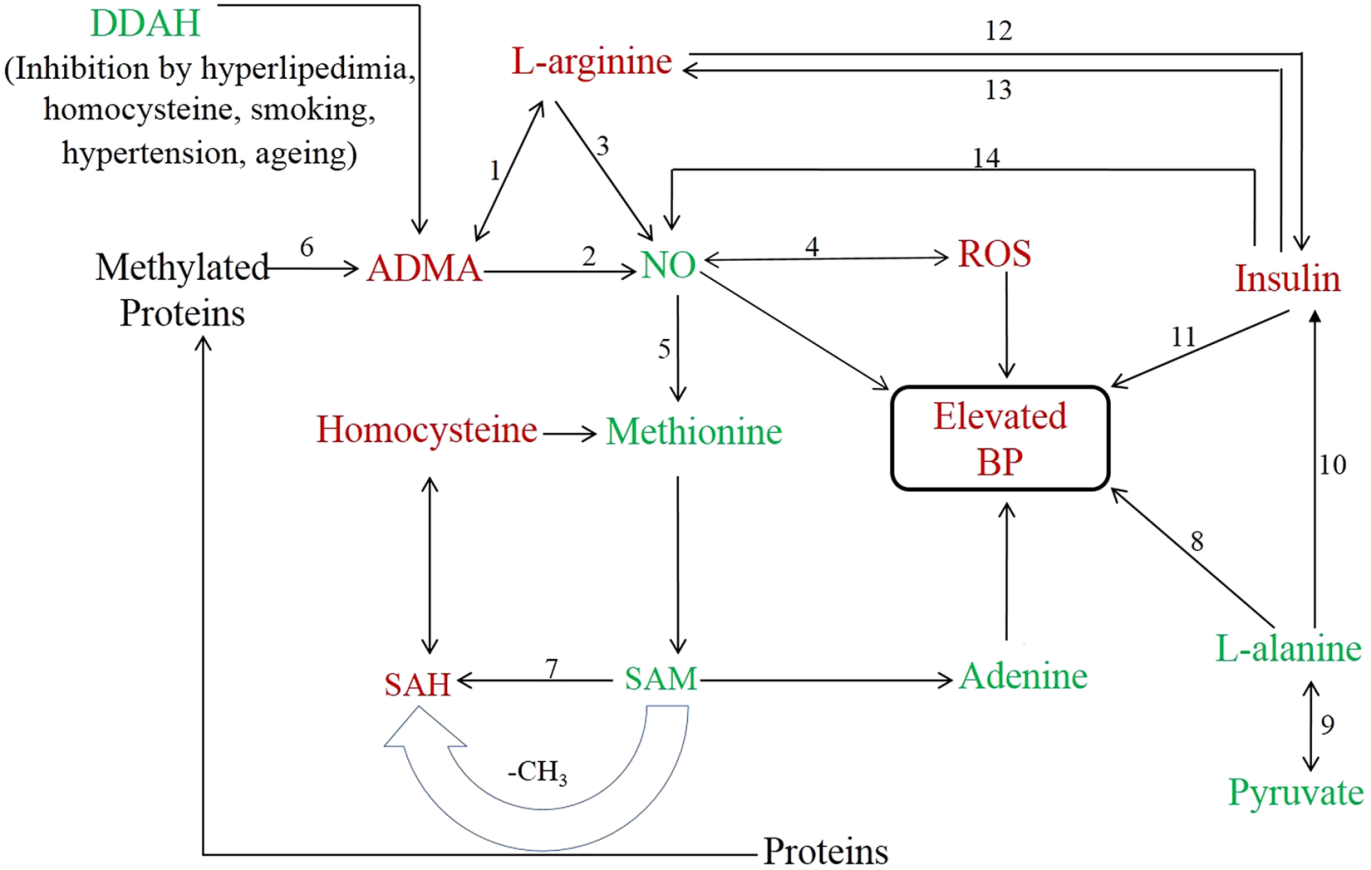
A flowchart representing possible dynamics of metabolites describing results and implications of elevated blood pressure (green colour represents decreased concentrations and red colour represents elevated concentration in EH milieu; DDAH; dimethylarginine dimethylamino-hydrolase, ADMA; asymmetrical dimethyl arginine, NO; Nitric oxide, NOS; NO synthase, PRMT; protein methyltransferase, ROS; reactive oxygen species, SAM; S-adenosylmethionine, SAH; S-adenosylhomocysteine). 1; competitive inhibition, 2; NOS inhibition, 3; Nitric oxide synthase, 4; peroxy nitrite formation, 5; decrease in methionine synthase, 6; hydrolysis, 7; PRMT, 8; decrease in norepinephrine release, 9; alanine aminotransaminase, 10; Mitochondrial metabolism is also activated by alanine resulting in increase in ATP/ADP ratio in cytosol, that triggers insulin exocytosis via increased cytosolic Ca^2+^ concentration, 11; insulin resistance, 12; insulin release, 13; arginine uptake, 14; increases NO via insulin signalling.

Figure 5 depicts a plausible dynamics of metabolites causing essential hypertension. Of note, many positive or negative feedback mechanisms related with arginine, NO, ADMA, insulin, ROS, ACE (angiotensin converting enzyme) operate and any alteration could result in elevated blood pressure. However, prime discussion is concerning ROS and ADMA resulting in decreased NO bioavailability. Also, adenine incorporation in place of guanine in angiotensinogen promoter is reported to be associated with essential hypertension^73^, thereby relating with decreased concentration of adenine in EH milieu of our NMR data.

Arginine, methionine, adenine, uracil, alanine, and pyruvate metabolites exhibit conspicuous peaks in NMR-derived metabolic profile, thus enabling quantitative assessment. This is particularly advantageous as compared to traditional biochemical methods where individual measurement is required using color formation reaction. Also, resultant compromised reproducibility needs to be addressed in conventional methods. In this study, simultaneous evaluation of metabolites in actual EH patients are revealing as metabolic signatures peculiar of EH. Moreover, the multivariate statistics enables a revelation in terms of dependent variables that create mutual effect to classify study subjects with high accuracy.

## Conclusions

The application of ^1^H NMR spectroscopy based metabolomics in EH patients undersores the use of this strategy to relate subtle metabolic differences with EH. It is speculated that the prediction model and linear regression presented here may serve as a surrogate method for screening of hypertension. With regard to the monitoring of blood pressure, a parallel gaze into the metabolome may enhance the interpretation of EH etiology. Similar studies with a large sample size would be appreciated to further validate the outcomes.

## Methods

### Study design

This is a case-control study. The study was approved by the ethics committee of King George’s Medical University (KGMU), and Sanjay Gandhi Post Graduate Institute of Medical Sciences (SGPGIMS) Lucknow, U.P. India. The patients and control subjects were recruited from the Department of Cardiology, KGMU, and the Department of Cardiology, SGPGIMS, Lucknow. All methods were performed in accordance with the relevant guideline and regulations. The recruitment of all participants in the study was done after a verbal and written explanation of study design and written informed consents were obtained.

### Patients and sample collection

A medical history and clinical parameters were obtained from all study subjects including age, blood pressure levels, presence of any definite cause of hypertension, serum creatinine and urea levels, lipid profile, Na^+^ and K^+^ levels, symptoms of hypertension and family history.

The inclusion criteria of the EH cohort comprised non-diabetic males between 35 to 50 years of age with stage I (Systolic BP = 140–159 mm Hg and/or Diastolic BP = 90–99 mm Hg) and stage II (SBP ≥ 160 mm Hg and/or DBP ≥ 100 mm Hg) hypertension of unknown etiology. Exclusion criteria of study subjects include the following; obese (BMI > 30, determined by weight and height), hyper-lipidemia (lipid profile, within one month), diabetes (evaluation of HbA1c), subjects on steroids for any medical condition, coronary artery disease (CAD), history of angina, or previous episode of MI, or any coronary intervention, valvular heart disease (by clinical evaluation of heart rhythms), stroke (history, previous treatment records, clinical evaluation), any previously reported renal hypertension case (history and treatment records), malignancies and auto-immune diseases (by symptomatic assessment and clinical evaluation, peripheral vascular disease, endocrine disorders.

A total of 96 patients presenting with a high blood pressure value, i.e., SBP > 140 mm Hg and/or DBP > 90 mm Hg were initially enrolled in the study. Patients with accelerated hypertension or with SBP > 180 mm Hg and/or DBP > 110 mm Hg were started on anti-hypertensives in the first instant and were excluded. On first presentation, out of 96 patients, 71 had stage I hypertension and 25 had stage II. Hemodynamically, out of 71 stage I patients, 38 patients had isolated diastolic blood pressure with normal SBP; the remaining 33 patients had combined systolic-diastolic blood pressure. All 25 stage II patients had combined systolic-diastolic BP. These patients were advised to follow a DASH (Dietary Approaches to Stop Hypertension) diet that is rich in green vegetables and fruits, low-fat or non-fat dairy and low in salt/sodium, maintain a healthy regimen, aerobic exercises, and to avoid tobacco/cigarette consumption. The patients were asked to maintain a BP chart for a week to rule out the possibility of white-coat hypertension/aggravation. Also, to search for any secondary cause of hypertension, the patients were advised to obtain for a set of biochemical tests including electrolyte levels (Na^+^, K^+^), glycosylated hemoglobin, blood urea, serum creatinine, lipid profile, hematocrite and urinalysis.

The patients were asked to visit the clinic after a week. In the follow-up week, the number of patients turning up decreased to 89. Out of these 89 patients, 25 were excluded based on one of the following reasons: perturbed biochemical test results or inconsistency in elevated blood pressure levels evident from a home-maintained BP chart. Wherever any conflict of opinion existed between attending physicians regarding the secondary cause of hypertension, the case was excluded. Thus, 64 EH patients were included in the study. Among these 64 patients, 45 had stage I hypertension and 19 had stage II. Among the 45 stage I hypertensive patients, 31 had isolated elevated DBP while 14 had combined elevated systolic-diastolic BP. All stage II EH patients had combined elevated systolic-diastolic BP. The stage I patients were followed up with life style modifications and stage II patients were started on anti-hypertensives at the discretion of the treating physician.

For the control group, 59 age comparable healthy volunteers were recruited from the same population region, subjects visiting the clinic for a routine examination or the patients’ blood relatives of same household (siblings). The control subjects were also advised regarding diet modifications and health regime similar to EH individuals. As high as 35.5% HC also had a family history of EH (pre-disposing them to a risk of EH) and about 52.5% HC also had a smoking habit prior to enrolment in the study. These aspects render the choice of EH patients and HC from among a representative population differing only in the chosen character (presence or absence of essential hypertension).

Blood samples (4.0 ml) were collected in vacutainer tubes via venous puncture in morning hours between 7.00 to 9.00 AM. To obtain serum, the samples were allowed to clot for about half an hour followed by centrifugation at 3000 g at 4 °C for 15 minutes.

#### Blood Pressure Measurement

A mercury sphygmomanometer with an adult-size cuff was used to measure the blood pressure. Blood pressure was measured twice at every clinic visit by trained staff after a 15 minute rest with the patient seated in a chair, with back supported and bare arm kept at heart level.

One of the major problems encountered with blood serum samples is the hindrance offered by the presence of proteins and lipoproteins that render the obtained NMR spectra unclear and often mask peaks of metabolites that exhibit smaller peaks. Conventionally, a Carr-Purcell-Meiboom-Gill pulse programming sequence is used to minimize these interferences based on suppression of resonances. In this study, we applied a mechanical cut-off to address the issue of signal attenuation of less-abundant metabolites. Centrifugal filters (3 kDa cutoff; Vivospin Turbo 4, VS04T91, Sartorius) were first washed with 1000 μl phosphate buffer in triple distilled water at 10,000 rpm for 30 min. Thereafter, a 1.5 ml serum sample from the study subject was poured into the centrifugal filter followed by centrifugation at 10,000 rpm at 4 °C for half an hour. The filtrates so obtained were collected in Eppendorf tubes and maintain the pH at 7.05 using 0.1 mol/l HCl or NaOH, if required, subsequently stored at -80 °C till further assayed. For all samples, the same protocol was followed.

### NMR Experiments

Prior to NMR acquisition, the filtered serum samples were thawed at 25 °C and 400 μL sample volume were transferred to 5 mm NMR tubes. All NMR experiments were conducted at 298 K using a Bruker Avance-III 800 MHz spectrometer containing a Z-shielded gradient and 5 mm broad-band inverse probe-head. Trimethylsilyl propionic acid sodium salt (TSP) (50 µL of 1.25 mmol/l) deuterated at CH2 group was employed for the deuterium lock, external reference, and standard signal for the quantification of metabolites. 1D ^1^H NMR experiments were performed for all samples by suppression of the water signal through pre-saturation. The parameters used were as follows: spectral width, 16666 Hz; time domain points, 65 k; relaxation delay, 10 s; pulse angle, 90°; number of scans, 128; and line broadening, 0.3 Hz. Bruker TopSpin software (version 2.1) was used for phase and baseline correction of all NMR data.

### Data Analysis

To facilitate data analysis, each NMR spectrum was aligned to the methyl peak of alanine (1.45 ppm, doublet) followed by generation of spectral bins. For this purpose, Bruker AMIX (Analysis of MIXtures), version 3.8.7, BioSpin, Germany, software was used. A total of 270 integral bins were generated from each NMR spectrum (0.5 to 8.5 ppm), each of width 0.03 ppm. The region between 4.7 to 5.1 ppm was eliminated to compensate for the residual water resonances. The data matrix so obtained was exported to Microsoft Excel 2010 and used for further analysis. Each integral point was divided by the sum of all integral points in a sample to perform normalization. This helped in minimizing variations in sample preparation and analysis. Multivariate statistical analysis, principal component analysis (PCA), and orthogonal partial least-squares discriminant analysis (OPLS-DA) were carried out to analyze metabolic profiles and examine scattering patterns of different cohorts using Unscrambler X software (version 10.0.1, Camo, Norway).

The loading plot obtained was used to identify the variables that created class-difference between the study groups. Retrospective assignment from NMR spectra led to the determination of significantly perturbed metabolites. Their measured coefficient values were also recorded.

To circumvent any mistakes in the mathematical model, a 7-fold internal cross-validation (ICV) was executed using a leave-one-out tactic. Based on Fisher and Yates tables, all samples were first randomly divided into two groups: 60% samples constituting the training set and the remaining 40% as the test set. To test the predictability of the method for the test set, OPLS-DA was again applied to the training set of samples. The rigorous ICV approach provided proof of model validation by providing values of explained variance (R^2^) and accuracy of prediction (Q^2^). Prophecies of the test samples were achieved using a Y-predicted scatter plot. Putative biomarkers were identified with the use of S-plots and the scores of variable importance plots (VIPs). To define the clinical significance and certify the robustness of the mathematical scoring-based classification to distinguish between EH and HC cohorts, receiver operating characteristic (ROC) curve analysis was executed. The relationship between systolic blood pressure values and diastolic blood pressure values with ^1^H NMR metabolomics-derived variables was also individually assessed using linear regression analysis.

## Electronic supplementary material

Supplementory information
