# Supplementary material for: Essential hypertension: A filtered serum based metabolomics study
Source: Sci Rep. 2017 May 19;7:2153. doi: 10.1038/s41598-017-02289-9 (PMC5438387; doi:10.1038/s41598-017-02289-9)
Supplement: Supplementary file 1 — Supplementory information [file 41598_2017_2289_MOESM1_ESM.doc]

**Essential hypertension: A filtered serum based metabolomics study**

Keerti Ameta^1^, Ashish Gupta^2*^, Sudeep Kumar^3^, Rishi Sethi^4^, Deepak Kumar^2^,

Abbas Ali Mahdi^1^

^1^Department of Biochemistry, King George’s Medical University, Lucknow,

^2^Centre of Biomedical Research, SGPGIMS Campus, Lucknow

^3^Department of Cardiology, SGPGIMS, Lucknow

^4^Department of Cardiology, King George’s Medical University, Lucknow

**Corresponding Authors:**

Ashish Gupta, PhD

Centre of Biomedical Research

SGPGIMS campus,

Raebareli Road,

Lucknow, 226014

UP. India

Phone: 91-522-2668700

Fax: 91-522-2668215

E-mail: ashishg24@yahoo.co.in


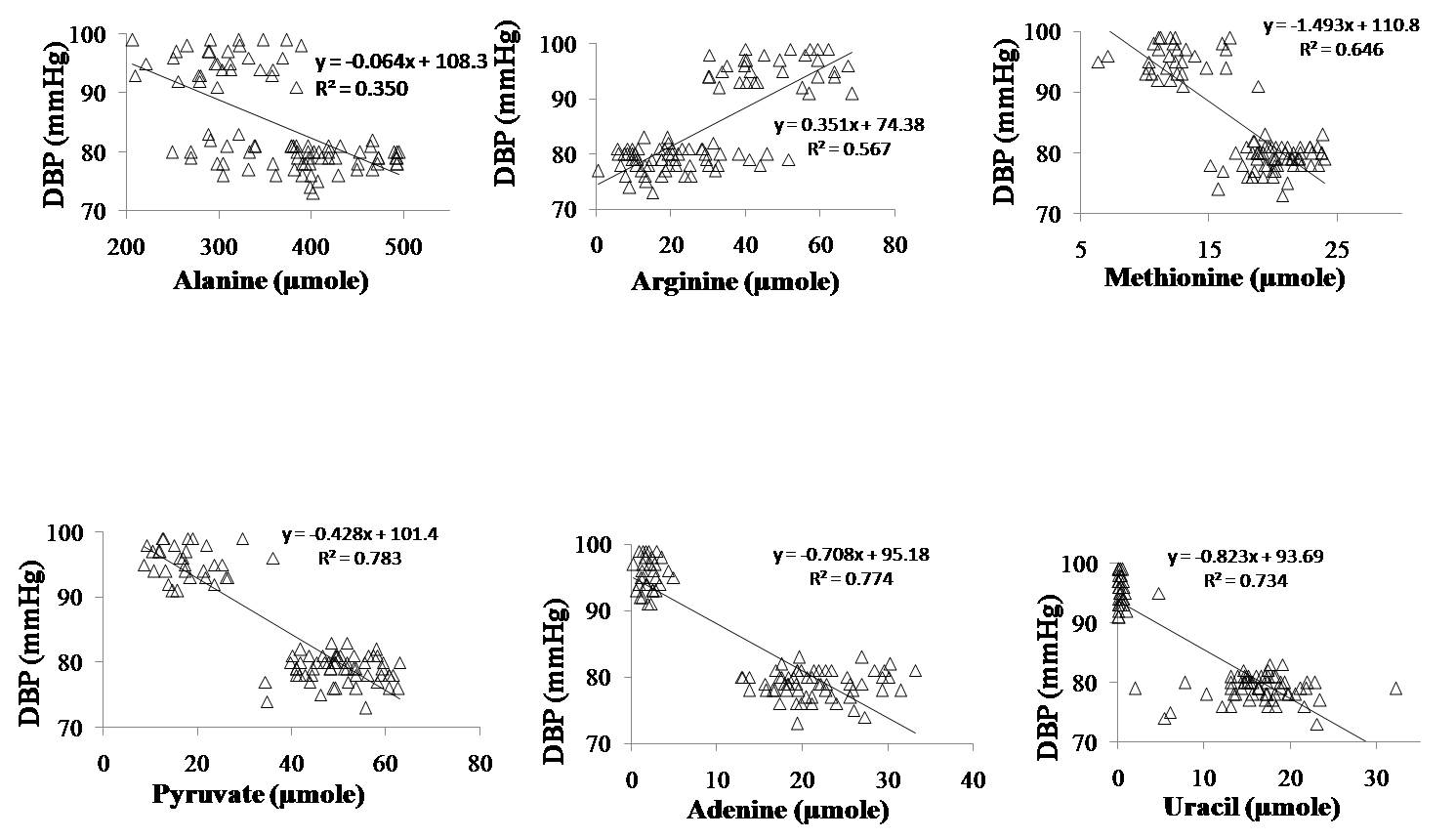


Figure S1: Regression analysis of serum metabolomics derived six biomarkers with diastolic blood pressure


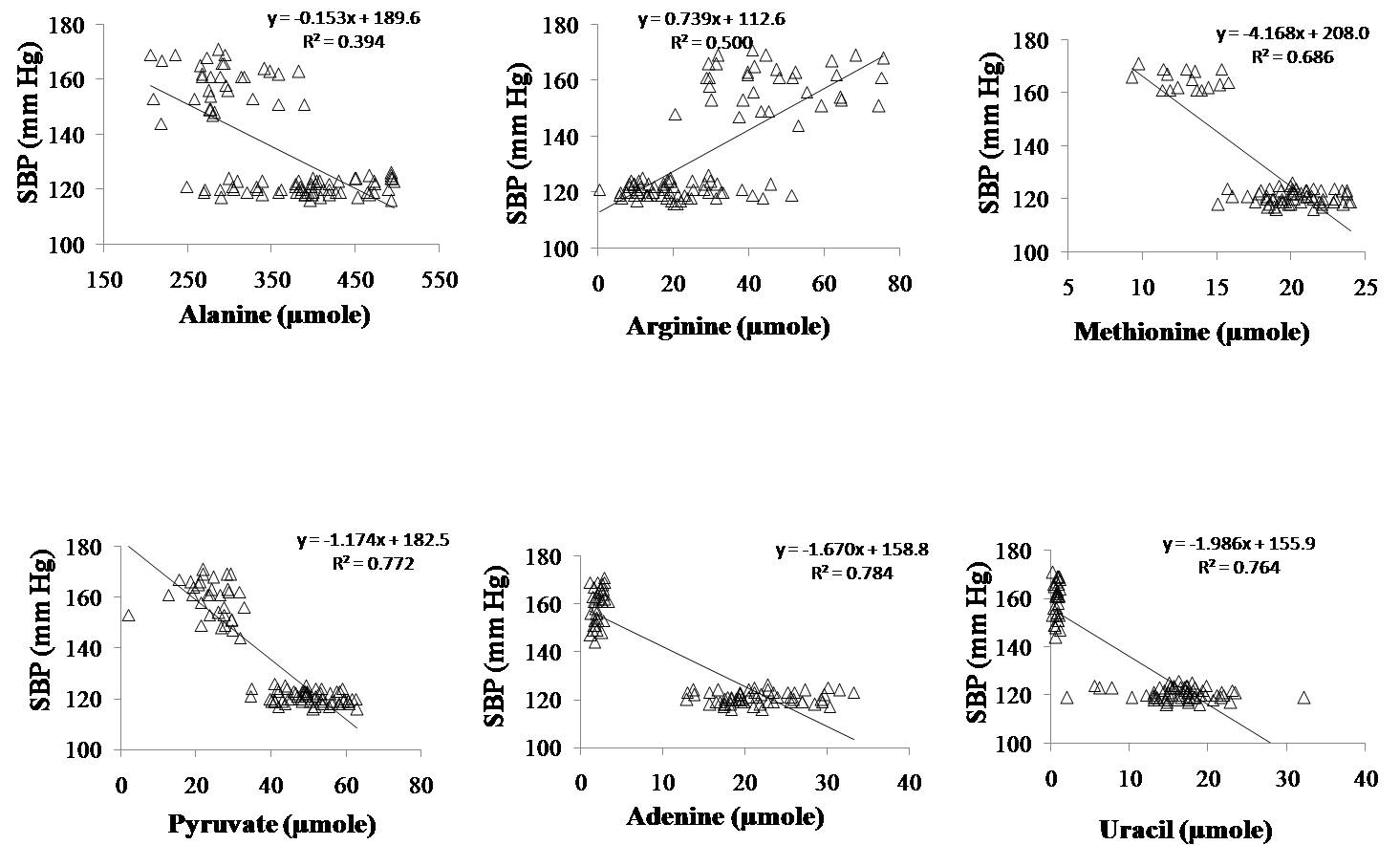


Figure S2: Linear regression analysis of serum metabolomics derived six biomarkers with combined Systolic blood pressure


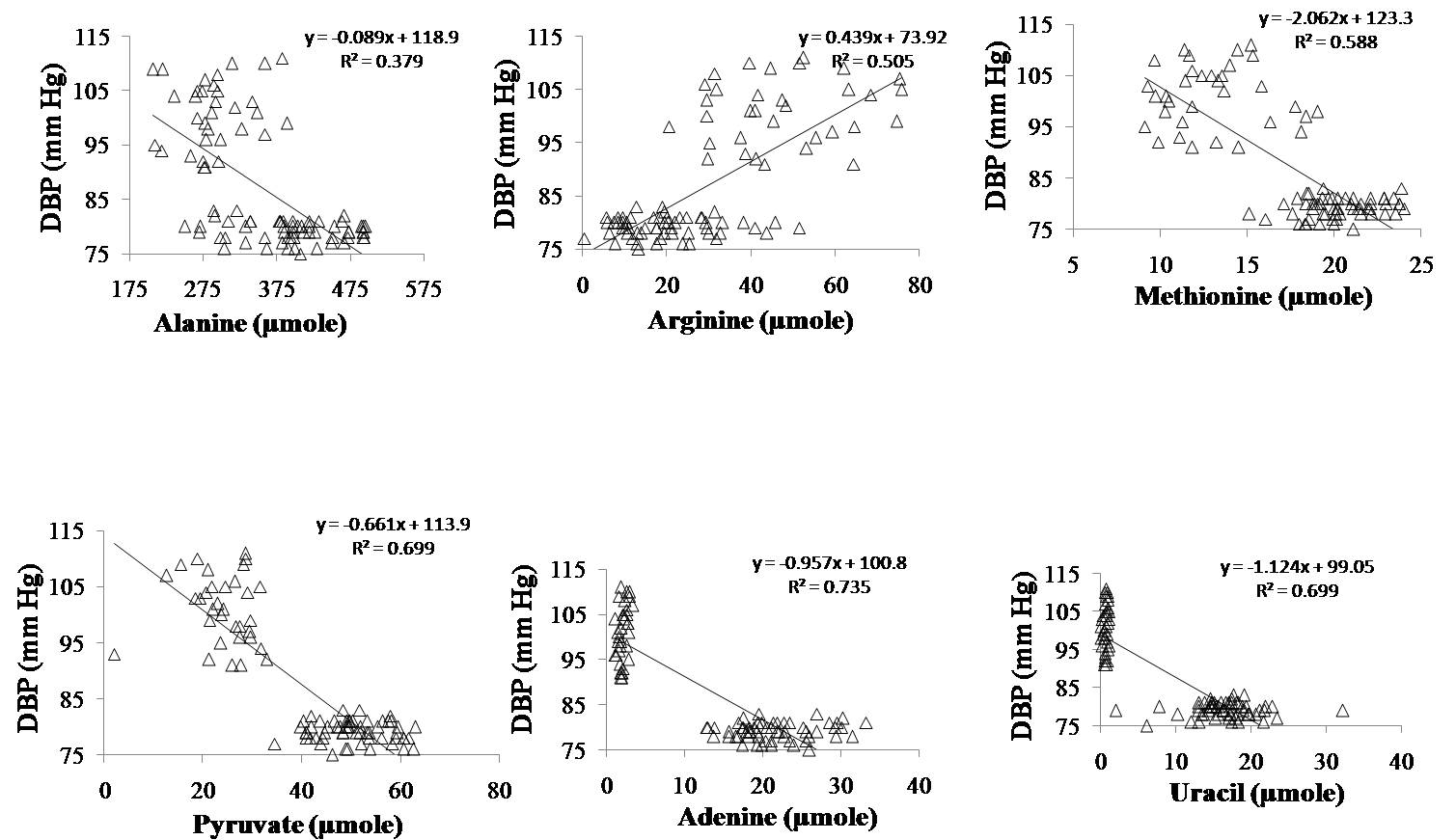


Figure S3: Linear regression analysis of serum metabolomics derived six biomarkers with combined diastolic blood pressure
